# Access and response to direct antiviral agents (DAA) in HIV-HCV co-infected patients in Italy: Data from the Icona cohort

**DOI:** 10.1371/journal.pone.0177402

**Published:** 2017-05-17

**Authors:** Antonella d'Arminio Monforte, Alessandro Cozzi-Lepri, Francesca Ceccherini-Silberstein, Andrea De Luca, Sergio Lo Caputo, Antonella Castagna, Cristina Mussini, Antonella Cingolani, Alessandro Tavelli, Milensu Shanyinde, Andrea Gori, Enrico Girardi, Massimo Andreoni, Andrea Antinori, Massimo Puoti, on behalf of Icona Foundation and HepaIcona Study Group

**Affiliations:** 1 Clinic of Infectious and Tropical Diseases, Department of Health Sciences, ASST Santi Paolo e Carlo, University of Milan, Milan, Italy; 2 Department of Infection and Population Health, Division of Population Health, UCL Medical School, Royal Free Campus, London, United Kingdom; 3 Department of Experimental Medicine and Surgery, University of Rome—Tor Vergata, Rome, Italy; 4 UOC of Infectious Diseases, Dipartimento di Biotecnologie Mediche, University of Siena, Siena, Italy; 5 UOC of Infectious Diseases, Policlinico di Bari, Bari, Italy; 6 Department of Infectious Diseases, San Raffaele Scientific Institute, University Vita-Salute San Raffaele, Milan, Italy; 7 Infectious Disease Clinic, Department of Medical and Surgical Sciences for Children & Adults, University of Modena and Reggio Emilia, Modena, Italy; 8 Institute of Clinical Infectious Diseases, Department of Public Health, Catholic University of Sacred Hearth, Rome, Italy; 9 Icona Foundation, Milan, Italy; 10 Division of Infectious Diseases, ASST Monza-Brianza- San Gerardo Hospital, University Milano-Bicocca, Monza, Italy; 11 Department of Epidemiology, National Institute for Infectious Diseases "Lazzaro Spallanzani", Rome, Italy; 12 Clinical Infectious Diseases, Department of Systems Medicine, University of Rome—Tor Vergata, Rome, Italy; 13 HIV/AIDS Department, National Institute for Infectious Diseases "Lazzaro Spallanzani", Rome, Italy; 14 Department of Infectious Diseases, ASST Grande Ospedale Metropolitano Niguarda, Milan, Italy; Chiba University, Graduate School of Medicine, JAPAN

## Abstract

**Background:**

Real-life data on access and response to direct antiviral agents (DAA) in HIV-HCV coinfected individuals are lacking.

**Methods:**

HCV viremic, HIV-positive patients from Icona and Hepaicona cohorts naïve to DAA by January 2013 were included. Access and predictors of starting DAA were evaluated. Switches of antiretroviral drugs at starting DAA were described. We calculated sustained virological response (SVR12) in those reaching 12 weeks after end-of-treatment (EOT), and defined treatment failure (TF) as discontinuation of DAA before EOT or non-SVR12. Statistical analyses included Kaplan-Meier curves, univariable and multivariable analyses evaluating predictors of access to DAA and of treatment outcome (non-SVR and TF).

**Results:**

2,607 patients included. During a median follow-up of 38 (IQR:30–41) months, 920 (35.3%) patients started DAA. Eligibility for reimbursement was the strongest predictor to access to treatment: 761/1,090 (69.8%) eligible and 159/1,517 (10.5%) non-eligible to DAA reimbursement. Older age, HIV-RNA≤50 copies/mL were associated to faster DAA initiation, higher CD4 count and HCV-genotype 3 with delayed DAA initiation in those eligible to DAA reimbursement. Up to 28% of patients (36% of those on ritonavir-boosted protease inhibitors, PI/r) underwent antiretroviral (ART) modification at DAA initiation. 545/595 (91.6%) patients reaching EOT achieved SVR12. Overall, TF occurred in 61/606 patients (10.1%), with 11 discontinuing DAA before EOT. Suboptimal DAA was the only independent predictor of both non-SVR12 (AHR 2.52, 95%CI:1.24–5.12) and TF (AHR: 2.19; 95%CI:1.13–4.22).

**Conclusions:**

Only 35.3% had access to HCV treatment. Despite excellent rates of SVR12 rates (91.6%), only 21% (545/2,607) of our HIV-HCV co-infected patients are cured.

## Introduction

The natural history of chronic Hepatitis C virus (HCV) infection has dramatically changed upon the introduction of Direct Antiviral Agents (DAA) regimens. In clinical trials settings these regimens have led to sustained virological response at 12 weeks (SVR12) after end of treatment (EOT) in up to 80–96% of cases. DAA response in HIV-HCV coinfected individuals are similar to HCV mono-infected ones [1–8]. A cure of HCV infection reduces the risk of liver cancer by 76% and of death by 50% [9]; theoretically, it could also reduce HCV transmission.

Nevertheless, universal access to anti-HCV treatment is still unreachable. Despite International recommendations that all HCV-infected persons should receive treatment [10–11], payers have responded to the high cost of HCV medications by instituting restrictive reimbursement policies [9]. In addition a gap between universal access and universal effective treatment remains, partly caused by the difference between the need to treat and the possibility of doing so [12]. In Italy, as in several European countries and in some states in USA, DAA are reimbursed only for patients with advanced liver disease or severe extra-hepatic HCV-related complications. Whether they actually initiate DAA or not remains to be evaluated [13].

In addition, introduction of reimbursement has varied in many countries by specific DAA: in Italy sofosbuvir (SOF) was reimbursed by the Italian Agency of the National Health System (AIFA) from December 2014, simeprevir (SIM) from February 2015, daclatasvir (DCL) from April 2015 and sofosbuvir+ledipasvir (SOF/LDV), ombitasvir+paritaprevir+ritonavir (2D) and dasabuvir (DSB) from May 2015. This may lead to use of suboptimal treatment schedules.

Concerning Human Immunodeficiency Virus (HIV)-HCV co-infected individuals, interactions with antiretroviral agents (ART) could occur, with possible toxicities or inadequate drugs levels [14]. Thus, pro active change of ART is often required before DAA, with possible negative consequences on adherence and on resistance to antiretrovirals. It is also unclear whether initiation of DAA is possible in most cases through temporary modification of the current ART, according to Guidelines [15,16]. Finally, results of the trials have to be confirmed, particularly in these individuals.

The main aim of this analysis was to evaluate the rate of access and response to DAA treatment in a HIV-HCV co-infected population seen for care in Italy. Issues related to the management of their HIV disease in relation to DAA treatment were also examined.

## Methods

Patients from HepaICONA and Icona Italian cohorts are included. HepaIcona is a cohort of HIV-HCV co-infected patients started on January 2013 to address issues related to access and response to DAA. The main inclusion criteria are to be ART-experienced, HIV-HCV coinfected and currently DAA-naïve with detectable HCV-RNA. This analysis includes also the subset of HIV-HCV co-infected patients enrolled in Icona (the Italian cohort of ART-naïve at enrolment patients) under active follow-up on January 1, 2013, with detectable HCV-RNA and DAA-naive. Details of Icona cohort have been described elsewhere [17]. All patients have given informed consent to participate the study and ethic committee approval from all participating centers was obtained for both cohorts (S1 Table).

Baseline for this analysis was the date of enrolment in the cohorts or January 1^st^, 2013, whichever was the latest. Socio-demographic factors were collected at baseline, HIV- and HCV-related factors have been collected at baseline and during follow-up: biochemistry, HCV-RNA, HCV-genotype, hepatic stiffness by transient elastography, anti-hepatitis drugs received before and after enrolment (including interferon-IFN-, PegIFN and ribavirin-RBV), ART regimens and reasons for discontinuation, all severe clinical events, including liver-related (variceal, gastro-intestinal bleeding, ascites, hepatic encephalopathy, hepatocellular carcinoma HCC). Suboptimal DAA (sDAA) was defined by rating recommendations for currently available options described in the 2016 EASL (European Association for the Study of the Liver) guidelines [10] i.e. therapies with pegylated IFN-a and ribavirin, with or without DAAs, such as telaprevir, boceprevir, sofosbuvir or simeprevir (if non genotype 4) or single DAA +ribavirin. AIFA eligibility criteria were to be F3-F4 or one of the following comorbidities: HCV-related lymphoma, symptomatic cryoglobulinemia, liver transplantation, HCC.

### Statistical analyses

Characteristics of the study population at baseline were described after stratification by the AIFA eligibility criteria. Differences in categorical factors were tested using the chi-square test and median values for continuous variables compared using the Wilcoxon test.

In people displaying AIFA criteria for DAA reimbursement, we estimated the median time to access to DAA from date of enrolment by Kaplan-Meier method. Factors independently associated with the probability of starting DAA were identified by log-rank test and proportional hazards Cox regression analysis. When data on stiffness by transient elastography were not reported we used the Fibrosis-4 (FIB-4) index to evaluate fibrosis and considered equivalent to F4 cases with FIB-4 >3.25 [18]. A sensitivity analysis was conducted using only data of participants with an available measure of stiffness. We also run a sensitivity analysis on access to DAA after left censoring the survival time at June, 1 2015 (date of availability of all currently used DAA in Italy).

In those who started DAA, we described the changes in ART regimen occurring in the previous 3 months before DAA. We calculated the median duration of DAA treatment in subgroups stratified by HCV-genotype, RBV use and decompensated cirrhosis and durations between groups was compared using the Wilcoxon signed rank test.

We included all those who started DAA and had 12 weeks data after EOT available to study the binary response (SVR12 yes/no) by logistic regression. We evaluated the rate of treatment discontinuation before EOT.

SVR12 was defined as a HCV-RNA result below the limit of detection at 12-weeks follow-up (SVR12) or thereafter. We defined treatment failure as a combined endpoint including treatment discontinuation or lack of SVR12 by intention-to-treat (ITT) switch = failure analysis.

Multivariable models have been constructed by including potential predictors in the models.

In those who started DAA and completed the treatment course we performed univariable and multivariable analyses of covariance (ANCOVA) on the absolute CD4 count (fitted in the raw scale) and HIV-RNA (log10 scale) to evaluate the potential effect of the inclusion of RBV in the DAA on these biomarkers.

## Results

### Characteristics of the HIV-HCV co-infected patients

A total of 2,607 HIV-HCV co-infected patients have been analysed using data frozen at December 15, 2016; 1,090 (41%) displayed AIFA criteria for DAA reimbursement. Table 1 shows the characteristics of the participants at baseline stratified in 2 groups: those eligible (n = 1,090) and those not eligible (n = 1,517) for DAA reimbursement.

**Table 1:** Characteristics of the whole cohort, according to eligibility to reimbursement of DAA.

Characteristics	Not eligible	Eligible	p-value	Total
	N = 1,517	N = 1,090		N = 2,607
***Age*, *years***				
Median (IQR)	49 (44, 53)	52 (49, 55)	< .001	50 (46, 54)
>50 years, n (%)	701 (46.2)	781 (71.7)	< .001	1482 (56.8)
***Gender*, *n (%)***			< .001	
Female	446 (29.4)	236 (21.7)		682 (26.2)
***Mode of HIV Transmission*, *n (%)***			< .001	
Heterosexual contacts	206 (13.6)	114 (10.5)		320 (12.3)
IDU	1052 (69.3)	839 (77.0)		1891 (72.5)
Homosexual contacts	169 (11.1)	44 (4.0)		213 (8.2)
Other/Unknown	90 (5.9)	93 (8.5)		183 (7.0)
***Nationality*, *n (%)***			< .001	
Not Italian	98 (6.5)	38 (3.5)		136 (5.2)
***Employment*, *n (%)***			0.002	
Unemployed	319 (21.0)	178 (16.3)		497 (19.1)
Employed	1050 (69.2)	824 (75.6)		1874 (71.9)
Other/unknown	148 (9.8)	88 (8.1)		236 (9.1)
***Hazardous drinking*, *n (%)***				
Yes	71 (6.3)	56 (8.2)		127 (7.0)
***BMI*, *Kg/m2***				
Median (IQR)	23 (21, 25)	24 (21, 27)	< .001	23 (21, 26)
>30, n (%)	52 (5.7)	51 (7.5)	0.131	103 (6.4)
***CD4 count nadir*, *cells/ mm3***				
Median (IQR)	197 (86, 301)	149 (66, 250)	< .001	177 (77, 283)
***CD4 count*, *cells/mm3***				
Median (IQR)	599 (415, 833)	490 (291, 738)	< .001	559 (360, 792)
***CD8 count*, *cells/mm3***				
Median (IQR)	897 (650, 1225)	760 (512, 1080)	< .001	848 (592, 1179)
***HIV-RNA*, *log10 copies/mL***				
Median (IQR)	1.3 (0.0, 1.6)	1.3 (0.0, 1.6)	0.003	1.3 (0.0, 1.6)
***Calendar year of HIV diagnosis***				
Median (IQR)	1996 (1989, 2005)	1991 (1987, 1999)	< .001	1994 (1987, 2003)
***Calendar year of first ART***				
Median (IQR)	2001 (1997, 2009)	1999 (1996, 2006)	< .001	2000 (1997, 2008)
***HCV-RNA*, *log10 IU/L***				
Median (IQR)	6.0 (5.4, 6.5)	6.0 (5.4, 6.5)	0.553	6.0 (5.4, 6.5)
***HCV-genotype*, *n (%)***			< .001	
1a	534 (35.2)	411 (37.7)		945 (36.2)
1b	178 (11.7)	125 (11.5)		303 (11.6)
1 not specified	52 (3.4)	46 (4.2)		98 (3.8)
2	44 (2.9)	26 (2.4)		70 (2.7)
3	324 (21.4)	301 (27.6)		625 (24.0)
4	257 (16.9)	149 (13.7)		406 (15.6)
Other/unknown	128 (8.4)	32 (2.9)		160 (6.1)
***ALT*, *IU/L***				
Median (IQR)	46 (30, 72)	65 (41, 108)	< .001	53 (33, 86)
>2 ULN, n(%)	334 (22.8)	437 (41.8)	< .001	771 (30.7)
***AST*, *IU/L***				
Median (IQR)	36 (27, 51)	64 (43, 99)	< .001	44 (30, 70)
>2 ULN, n (%)	122 (8.6)	338 (32.9)	< .001	460 (18.8)
***Bilirubin*, *IU/L***				
Median (IQR)	0.6 (0.4, 1.0)	0.8 (0.5, 1.5)	< .001	0.6 (0.4, 1.2)
***Gamma-GT*, *IU/L***				
Median (IQR)	54 (30, 105)	92 (53, 171)	< .001	69 (37, 128)
>2 ULN (%)	413 (30.8)	450 (51.3)	< .001	863 (38.9)
***Platelets*, *x 10***^***9***^***/L***				
Median (IQR)	196 (162, 240)	130 (86, 174)	< .001	173 (128, 220)
>150 n (%)	1221 (82.9)	383 (36.4)	< .001	1604 (63.6)
***Stiffness*,**				
Median (IQR)	6 (5, 8)	16 (12, 25)	< .001	9 (6, 15)
0–7 Kpa (%)	501 (66.2)	18 (2.5)	< .001	519 (34.9)
7–10 Kpa (%)	256 (33.8)	39 (5.3)		295 (19.8)
10+ Kpa (%)	0 (0.0)	673 (92.2)		673 (45.3)
***Fib-4***				
Median (IQR)	1.35 (0.99, 1.87)	3.55 (2.07, 5.72)	< .001	1.78 (1.19, 3.07)
0–1.45 (%)	799 (56.4)	117 (11.4)	< .001	916 (37.5)
1.45–3.25 (%)	617 (43.6)	340 (33.2)		957 (39.2)
3.25+ (%)	0 (0.0)	568 (55.4)		568 (23.3)
***MELD score***				
Median (IQR)	7.0 (6.4, 8.3)	8.1 (7.0, 10.4)	< .001	7.4 (6.4, 9.2)
***Decompensated cirrhosis*, *n (%)***				
Yes	0 (0.0)	116 (10.6)		116 (4.4)
***Hepatocarcinoma*, *n (%)***			< .001	
Yes	0 (0.0)	26 (2.4)		26 (1.0)
***Liver transplant*, *n (%)***			< .001	
Yes	0 (0.0)	17 (1.6)		17 (0.7)
***Other organ transplant*, *n (%)***			0.018	
Yes	0 (0.0)	4 (0.4)		4 (0.2)
***Lymphoma*, *n (%)***			0.002	
Yes	0 (0.0)	7 (0.6)		7 (0.3)
***Diabetes*, *n (%)***			< .001	
Yes	54 (3.6)	84 (7.7)		138 (5.3)
***Creatinine***				
Median (IQR)	0.8 (0.7, 1.0)	0.8 (0.7, 1.0)	0.456	0.8 (0.7, 1.0)
***eGFR*, *mL/min/1*.*73m^2^***				
Median (IQR)	100.6(86.5, 107.8)	100.0 (84.7, 106.8)	0.035	100.3 (85.7, 107.4)
0–60, ***n (%)***	67 (4.6)	59 (5.8)	0.408	126 (5.1)
60–90, ***n (%)***	378 (25.8)	265 (25.8)		643 (25.8)
90+, ***n (%)***	1021 (69.6)	702 (68.4)		1723 (69.1)
***Previous failure of HCV treatment*, *n (%)***				
Yes	360 (23.7)	405 (37.2)	< .001	765 (29.3)
Use of RBV	82 (51.6)	403 (53.0)	< .001	485 (52.7)
***Site geographical position*, *n (%)***			0.001	
North	419 (27.6)	345 (31.7)		764 (29.3)
Center	868 (57.3)	627 (57.6)		1495 (57.4)
South	229 (15.1)	116 (10.7)		345 (13.2)
***Started DAA*, *n (%)***				
Yes	159 (10.5)	761 (69.8)	< .001	920 (35.3)
***Sub-optimal DAA*, *n (%)***	17 (10.7)	17 (10.7)	0.559	1 (12.1)
Calendar year of starting DAA				
Median (IQR)	2015 (2015–216)	2015 (2015–2016)	0.420	2015 (2015–2016)
***Cohort*, *n (%)***				
HepaIcona	895 (59.0)	825 (75.7)	< .001	1720 (66.0)

IDU = intravenous drug user.

Fib-4 = fibrosis-4 score.

MELD = Model for End-Stage Liver Disease.

p-value: Chi-square or Kruskal-Wallis test as appropriate.

Hazardous drinking: For men is defined as >3 standard drinks per day and ≥6 drinks per occasion. For women defined as >2 drink per day and ≥5 drinks per occasion (https://www.niaaa.nih.gov/alcohol-health/overview-alcohol-consumption/alcohol-use-disorders).

In addition to the expected difference in terms of stage of fibrosis, previous failure to HCV therapy, and comorbidities, eligible patients showed different demographic features: they were older, less frequently females, more frequently infected through intravenous drug addiction, less frequently non-Italian, less frequently unemployed. Further, they had a longer history of HIV infection as documented by year of diagnosis, year of starting ART, CD4-positive lymphocyte (CD4) nadir (Table 1).

During a median follow-up of 38 (30–41) months, 761 (69.8%) patients eligible to reimbursement started DAA, and a further 159 (10.5%) not eligible, with F1-F2, started DAA within compassionate use programs.

Median calendar year of starting DAA was 2015 (2015–2016); 94 (12.4%) eligible and 17 (10.7%) non-eligible started suboptimal DAA regimens according to EASL [10].

#### Rate of access to DAA

**Fig 1:**
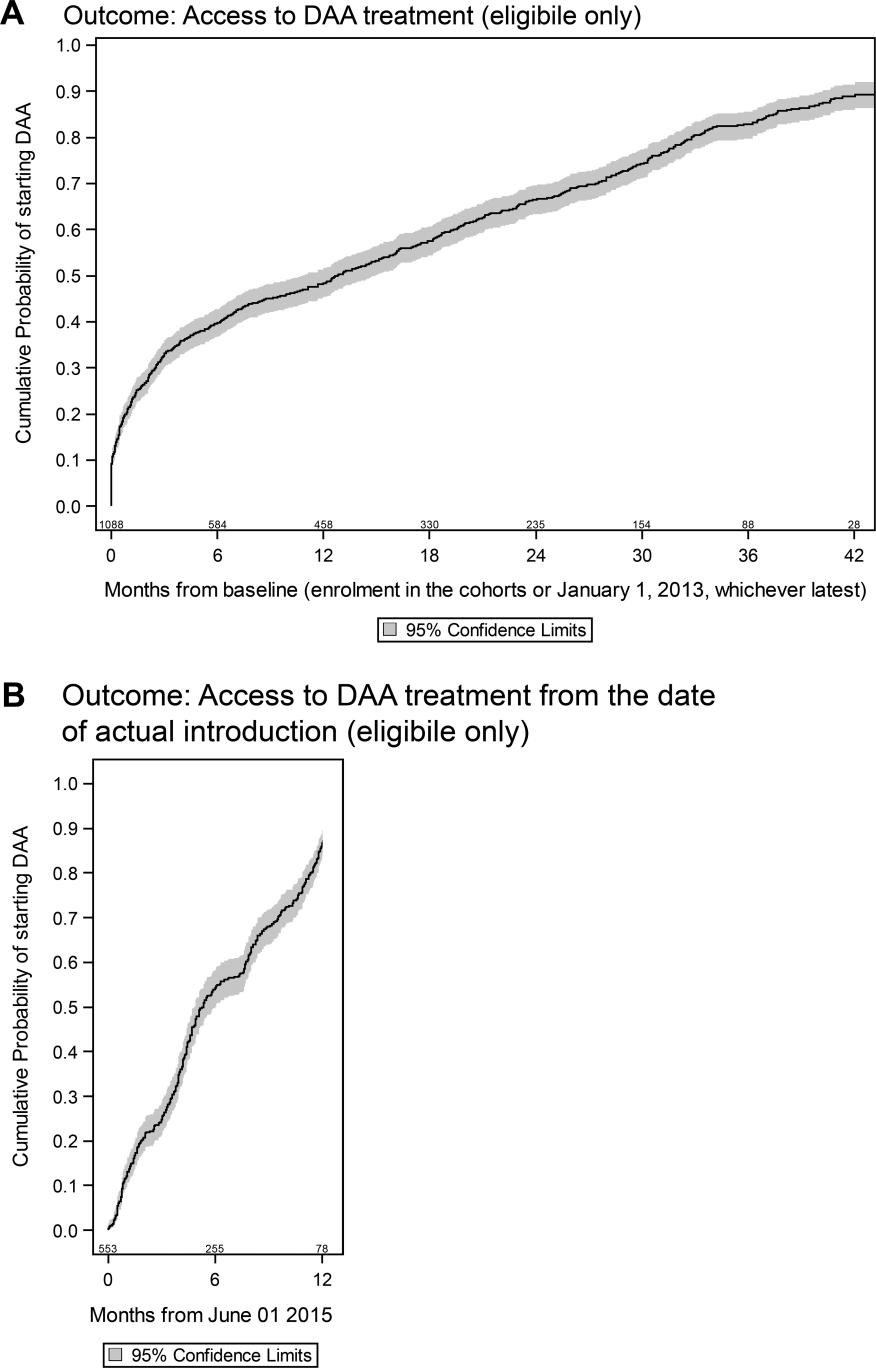
Kaplan-Meier estimate of the rate of access to DAA treatment. (A) From Baseline. (B) From June 2015.

In the 1,090 patients eligible to DAA reimbursement, the median time to DAA initiation was 12.8 (95% CI:10.8–15.0) months and by 42 months the probability of starting DAA was of 89% (95% CI: 86–92%) (Fig 1A). Nonetheless, the DAA uptake appeared to be gradual with 48% (95% CI:45–51%) starting by 12 months and 67% (95% CI: 64–70%) by 24 months from baseline, consistent with the delay in availability of interferon-free DAA regimens in Italy. When we restricted the analyses to June 2015, date of reimbursement of second-generation DAAs in Italy, the 1-year probability of starting DAA among patients eligible to reimbursement raised to 86.4% (95% CI: 83.5–89.3) (Fig 1B).

A number of factors were independently associated with the probability of starting DAA among patients eligible for reimbursement in the multivariable model (Table 2). Older age, having a HIV-RNA≤50 copies/mL were associated with earlier DAA initiation; previous unsuccessful treatment with IFN were marginally associated (Adjusted HR: 1.17; 95%CI: 0.98–1.40; p = 0.08), whereas higher CD4 count was associated with delayed DAA initiation. Patients with HCV genotype 3 had slower access to treatment as compared to those with genotype 1a. Similar results were obtained after restricting to participants for whom fibrosis was assessed by transient elastography (S2 Table). Previous unsuccessful treatment with IFN was associated to higher probability of starting DAA also after June 2015 (S3 Table).

**Table 2:** Relative hazards of starting DAA from fitting a Cox regression model.

	Relative hazards of starting DAA			
	Unadjusted RH (95% CI)	p-value	Adjusted HR (95% CI)	p-value
***Age*, *years***				
>50 years vs. below	1.5 (1.3, 1.8)	< .001	1.5 (1.2, 1.8)	< .001
***Gender***				
Female vs. Male	1.0 (0.9, 1.2)	0.8	1.1 (0.9, 1.4)	0.2
***Mode of HIV Transmission***				
Heterosexual contacts	1.0		1.0	
IDU	1.1 (0.9, 1.4)	0.5	1.0 (0.8, 1.4)	0.7
Homosexual contacts	1.2 (0.8, 1.8)	0.5	1.3 (0.8, 2.1)	0.3
Other/Unknown	1.1 (0.8, 1.6)	0.4	1.2 (0.8, 1.8)	0.3
***Employment***				
Unemployed	1.0		1.0	
Employed	1.2 (1.0, 1.5)	0.1	1.1 (0.9, 1.5)	0.4
Other/unknown	1.1 (0.8, 1.5)	0.6	1.1 (0.7, 1.5)	0.8
***CD4 count*, *cells/mm3***				
per 100 higher	1.0 (1.0, 1.0)	0.5	1.0 (0.9, 1.00)	0.04
***HIV-RNA*, *copies/mL***				
0–50 vs. >50	1.2 (1.0, 1.4)	0.1	1.6 (1.2, 2.1)	< .001
***Time from HIV diagnosis*, *years***				
per 10 longer	0.9 (0.8, 1.0)	0.02	0.9 (0.8, 1.0)	0.2
***HCV genotype***				
1a	1.0		1.0	
1b	0.8 (0.6, 1.0)	0.07	0.8 (0.6, 1.1)	0.1
2	0.7 (0.4, 1.1)	0.15	0.6 (0.3, 1.1)	0.09
3	0.8 (0.7, 1.0)	0.04	0.7 (0.6, 0.9)	0.008
4	1.0 (0.8, 1.3)	0.7	1.0 (0.8, 1.3)	0.7
Other/unknown	0.6 (0.3, 1.0)	0.03	0.6 (0.3, 1.2)	0.1
***HCV-RNA*, *log10 IU/L***				
per log higher	1.0 (0.9, 1.0)	0.4	1.0 (0.9, 1.1)	0.7
***Fib4***				
0–1.45	1.0		1.0	
1.46–3.25	1.2 (0.9, 1.5)	0.2	1.0 (0.8, 1.4)	0.9
3.25+	1.0 (0.7, 1.2)	0.7	0.7 (0.5, 1.1)	0.1
***Decompensated cirrhosis***				
Yes vs. No	1.0 (0.8, 1.2)	0.9	0.9 (0.7, 1.3)	0.7
***Diabetes***				
Yes vs. No	1.1 (0.9, 1.4)	0.4	1.1 (0.8, 1.5)	0.4
***Platelets*, *x 10***^***9***^***/L***				
>150 vs. below	0.9 (0.8, 1.1)	0.4	0.9 (0.7, 1.1)	0.2
***ALT*, *IU/L***				
>2 ULN vs. below	1.1 (0.9, 1.2)	0.4	1.1 (0.9, 1.4)	0.2
***Bilirubin*, *IU/L***				
per 10 higher	1.0 (0.4, 2.1)	0.9	1.0 (0.4, 2.4)	0.9
***eGFR*, *mL/min/1*.*73m^2^***				
90+	1.0		1.0	
60–90	1.1 (0.9, 1.3)	0.3	0.9 (0.6, 1.3)	0.6
0–60	0.8 (0.6, 1.2)	0.3	1.1 (0.9, 1.3)	0.6
***Previous failure of HCV treatment***				
Yes vs. No	1.2 (1.1, 1.4)	0.004	1.2 (1.0, 1.4)	0.1

Adjusted HR: adjusted for all factors examined in table and stratified by cohort.

### Patients who started DAA

**Fig 2:**
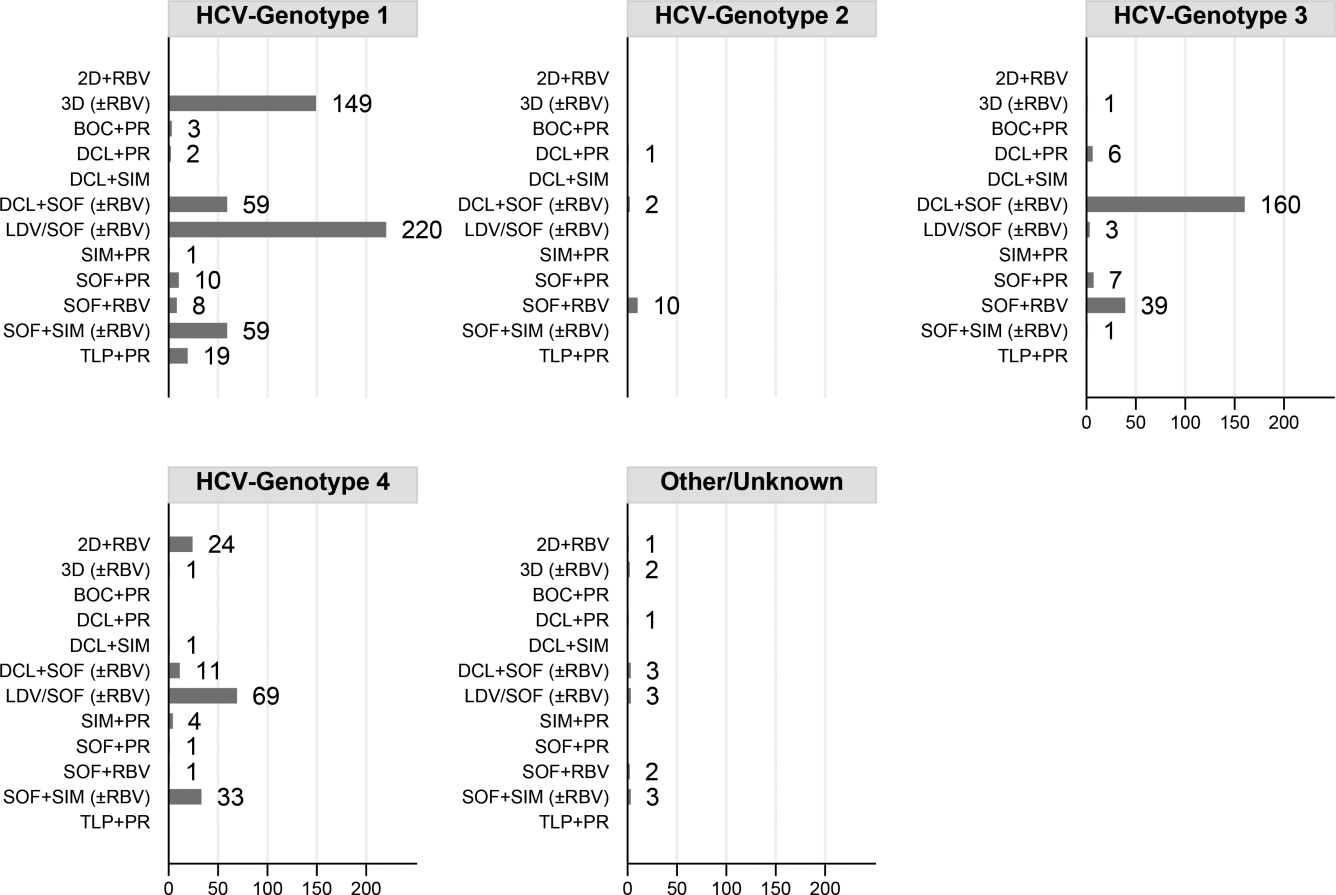
Distribution of DAA regimens by HCV genotype. 2D (ombitasvir,paritaprevir,ritonavir); 3D (ombitasvir,paritaprevir,ritonavir,dasabuvir);BOC (boceprevir);DCL (daclatasvir);LDV/SOF (ledipasvir,sofosbuvir);PR (peg-interferon,ribavirin);RBV (ribavirin);SIM (simeprevir);SOF (sofosbuvir);TLP (telaprevir).

A total of 920 patients started DAA. Fig 2 shows the distribution of DAA regimens according to genotypes. SOF/LDV±RBV was the most frequent regimen in genotype-1 patients (220, 42%), followed by 3D±RBV (149, 28%). SOF+DCL±RBV was the most frequent regimen in genotype-3 patients (160, 74%) and SOF+LDV±RBV was the most frequent one in genotype-4 patients (69; 48%). Overall, RBV was prescribed to 485 (53%) patients: 302/530 (57.0%) genotype-1, 1/13 (7.7%) genotype-2, 109/217 (50.2%) genotype-3, 63/145 (43.4%) genotype-4 and 10/15 (66.7%) other/unknown genotype (p<0.001).

A total of 79/142 (55.6%) patients with decompensated cirrhosis or HCC initiated DAA, with median Model For End Stage Liver Disease (MELD) score of 10.4 (IQR: 8.6–13.3).

DAA duration according to different characteristics is shown in Table 3. Subjects receiving RBV were treated for similar periods as compared to those not given RBV; subjects harbouring genotype 3 had a longer median duration of treatment than those harbouring other genotypes; treatment was longer for people with decompensated cirrhosis, and differed according to DAA regimen type.

**Table 3:** Duration of DAA according to the presence of ribavirin (RBV), decompensated cirrhosis, genotype and DAA regimen.

	Duration of DAA, weeks				
	Median (IQR)	p-value	< = 12 n (%)	>12 n (%)	p-value
***Use of Ribavirine***		0.431			0.932
No	13.5 (12.0, 24.0)		134 (37.2)	226 (62.8)	
Yes	12.7 (12.0, 24.0)		155 (37.7)	256 (62.3)	
***HCV genotype***		< .001			< .001
1	12.3 (12.0, 23.9)		159 (44.7)	197 (55.3)	
2	16.0 (12.0, 24.0)		3 (33.3)	6 (66.7)	
3	24.0 (23.7, 24.1)		15 (12.7)	103 (87.3)	
4	12.0 (12.0, 23.4)		54 (52.9)	48 (47.1)	
Other	12.0 (12.0, 22.0)		3 (60.0)	2 (40.0)	
1,2,4,other	12.1 (12.0, 23.9)		219 (46.4)	253 (53.6)	
***Decompensated cirrhosis***		< .001			< .001
No	12.3 (12.0, 24.0)		224 (42.1)	308 (57.9)	
Yes	23.9 (12.6, 24.3)		10 (17.2)	48 (82.8)	
***Regimen***		< .001			< .001
2D+RBV	12.2 (12.0, 24.0)		8 (40.0%)	12 (60.0%)	
3D(±RBV)	12.1 (12.0, 13.1)		63 (48.8)	66 (51.2)	
DCL+SOF(±RBV)	24.0 (23.4, 24.3)		15 (13.2)	99 (86.8)	
LDV/SOF(±RBV)	12.0 (12.0, 24.0)		73 (50.3)	72 (49.7)	
SOF+PR	12.4 (12.3, 14.6)		3 (16.7)	15 (83.3)	
SOF+RBV	24.0 (23.7, 24.4)		5 (11.6)	38 (88.4)	
SOF+SIM(±RBV)	12.0 (12.0, 12.1)		65 (72.2)	25 (27.8)	
TLP+PR	48.0 (26.9, 49.0)		1 (6.7)	14 (93.3)	

p-value: Wilcoxon or Kruskal-Wallis test or Chi-square test as appropriate.

CD4 cell counts and HIV-RNA copy levels variations from DAA initiation to EOT and 12 weeks after EOT are summarized in Table 4. Mean CD4 at starting DAA was 523 cells/mmc (Standard deviation-SD 216) and mean HIV-RNA log10 copies/ml was 1 (SD 1.07). Splitting the data according to intake of RBV, we observed a significantly higher decrease of mean CD4 counts at EOT in those receiving compared to those not receiving RBV; the effect of RBV on CD4 counts reversed by 12 weeks after EOT, when CD4 counts returned to pre-DAA levels. The percentage of patients with unquantifiable (< = 1 copies/mL) HIV-RNA varied from 49.6% at starting DAA to 65.8% at EOT and to 65.1% at 12 weeks after treatment withdrawal, with no differences according to RBV use (Table 4). Pre-treatment switch of ART and non-SVR had no effect on CD4 counts or HIV-RNA levels (data not shown).

**Table 4:** CD4 counts and HIV-RNA levels at EOT and 12 weeks after EOT from fitting an analysis of covariance model controlling for baseline values, according to use of ribavirin (RBV).

HIV lab markers	RBV in DAA	RBV-free DAA		RBV in DAA	RBV-free DAA	
	Unadjusted Mean (95% CI)		p-value	Adjusted Mean (95% CI)		p-value
***CD4 count at EOT***						
cells/mm3	481 (454, 507)	616 (588, 644)	< .001	480 (453, 507)	616 (588, 644)	< .001
***CD4 count 12 weeks after EOT***						
cells/mm3	623 (589, 656)	663 (628, 699)	0.1	624 (590, 657)	665 (629, 700)	0.1
***HIV-RNA at EOT***						
log10 copies/mL	0.5 (0.4, 0.6)	0.7 (0.5, 0.8)	0.07	0.5 (0.4, 0.6)	0.7 (0.6, 0.8)	0.03
***HIV-RNA 12 weeks after EOT***						
log10 copies/mL	0.6 (0.4, 0.7)	0.6 (0.5, 0.8)	0.4	1.0 (0.4, 0.7)	0.6 (0.5, 0.8)	0.4

Adjusted mean: adjusted for gender, age, HCV genotype, decompensate cirrhosis and diabetes.

HIV-RNA also adjusted for CD4 count at DAA initiation and vice versa.

### DAA and ART

In 829 (90%) of the 920 who started DAA, we had information on whether there has been a change in ART at DAA initiation as compared to 3 months prior to starting. A total of 230/829 (28%) underwent a modification of the third drug before DAA: atazanavir-ritonavir was replaced in 36/145 (25%) of cases, darunavir-ritonavir (DRV/r) in 40/117 (34%), rilpivirine (RPV) in 10/86 (12%), efavirenz in 36/75 (48%), raltegravir (RAL) in 6/221 (3%), elvitegravir in 8/17 (47%), 0/34 in dolutegravir (DTG).

A total of 118/328 (36.0%) participants receiving a ritonavir-boosted protease inhibitor (PI/r)-based regimen 3 months prior to DAA initiation were switched to an integrase inhibitor (INI) (n = 113) or RPV (n = 5); 36/175 (21%) patients on a non nucleoside reverse transcriptase inhibitor (NNRTI)-based regimen were switched to an INI-based regimen (n = 30) or to other classes (n = 6). Only 5/272 (2%) patients receiving an INI-including regimen changed the third drug class (4 into RPV, one to DRV/r).

### Response to DAA

**Fig 3:**
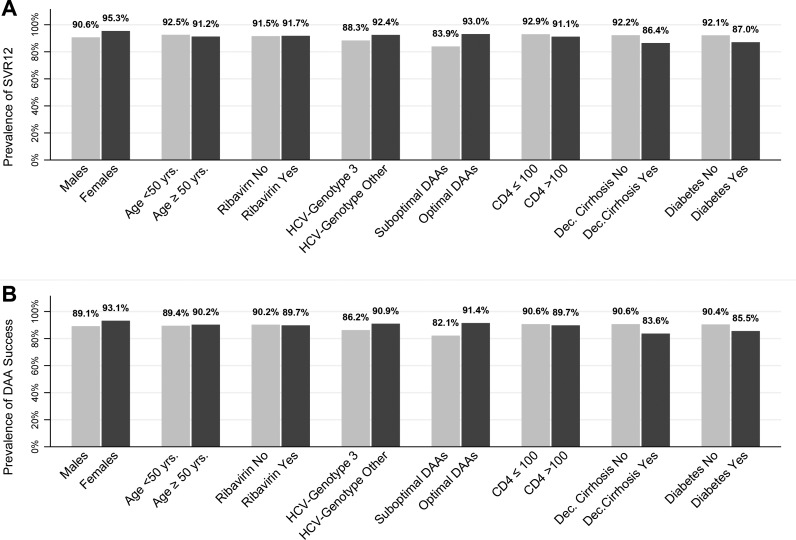
Response to DAA according to different conditions. (A) Prevalence of SVR12. (B) Prevalence of DAA success.

A total of 595 patients (65.9%) of the 920 who started DAA reached the SVR follow-up time while still on DAA and week 12 HCV-RNA value was recorded; 545 (91.6%) experienced SVR and 50 (8.4%) virological failure (non-SVR12). SVR12 was reached in 106/120 (88.3%) of genotype 3 (92% if treated with SOF+DCL+RBV for 24 weeks) and 439/475 (92.4%) of patients with genotype other than 3 (p = 0.15); in 297/324 (91.7%) patients treated with RBV and 248/271 (91.5%) starting a RBV-free regimen (p = 0.95); in 51/59 (86.4%) patients with decompensated cirrhosis and in 494/536 (92.2%) without (p = 0.04, Fig 3A). After adjustment for age, gender, HCV-genotype, RBV, nadir CD4, diabetes, and decompensated cirrhosis, the only variable independently associated with non-SVR12 was the use of suboptimal DAA (HR 2.52; 95%CI 1.24–5.12 vs optimal DAA). People with decompensated cirrhosis remained with a nearly 2-fold higher risk of failure although short of statistical significance (OR: 1.79; 95%CI: 0.77–4.16; p = 0.17) (Table 5A).

**Table 5:** Odds ratios of A) virological failure (non-SVR12). B) treatment failure (TF) from fitting a logistic regression model.

**A) non-SVR12**			**OR of DAA failure from fitting a logistic regression model**			
**Characteristics**	**Virological failure**	**SVR**	**Unadjusted OR (95% CI)**	**p-value**	**Adjusted OR (95% CI)**	**p-value**
	N = 50	N = 545				
***Age*, *years*, *n (%)***						
0–50	13 (26.0)	160 (29.4)	1.0		1.0	
51+	37 (74.0)	385 (70.6)	1.0 (0.5, 2.1)	0.9	1.0 (0.5, 2.0)	0.9
***Gender*, *n (%)***						
Male	44 (88.0)	424 (77.8)	1.0		1.0	
Female	6 (12.0)	121 (22.2)	0.5 (0.2, 1.1)	0.1	0.5 (0.2, 1.2)	0.1
***Ribavirin*, *n (%)***						
Not used	23 (46.0)	248 (45.5)	1.0		1.0	
Used	27 (54.0)	297 (54.5)	1.0 (0.5, 1.7)	0.9	1.0 (0.6, 1.8)	1.0
***HCV Genotype*, *n (%)***						
1,2,4,other	36 (72.0)	439 (80.6)	1.0		1.0	
3	14 (28.0)	106 (19.4)	1.6 (0.8, 3.1)	0.1	1.2 (0.6, 2.4)	0.7
*Sub-optimal DAA*, *n (%)*						
No	35 (70.0)	467 (85.7)	1.0		1.0	
Yes	15 (30.0)	78 (14.3)	2.6 (1.3, 4.9)	0.005	2.5 (1.2, 5.1)	0.011
***CD4 count nadir*, *cells/mm3*, *n (%)***						
0–100	11 (22.0)	144 (26.4)	1.0		1.0	
101+	39 (78.0%	401 (73.6)	0.79 (0.39, 1.57)	0	0.8 (0.4, 1.6)	0.537
***Decompensated cirrhosis*, *n (%)***						
No	42 (84.0)	494 (90.6)	1.00		1.0	
Yes	8 (16.0)	51 (9.4)	1.8 (0.8, 4.1)	0.1	1.8 (0.8, 4.2)	0.2
***Diabetes*, *n (%)***						
No	43 (86.0)	498 (91.4)	1.0		1.0	
Yes	7 (14.0)	47 (8.6)	1.7 (0.7, 4.0)	0.2	1.5 (0.6, 3.7)	0.4
**B) TF**			**OR of DAA failure from fitting a logistic regression model**			
**Characteristics**	**Treatment failure**	**SVR**	**Unadjusted OR (95% CI)**	**p-value**	**Adjusted OR (95% CI)**	**p-value**
	N = 61	N = 545				
***Age*, *years*, *n (%)***						
0–50	19 (31.1)	160 (29.4)	1.0		1.0	
51+	42 (68.9)	385 (70.6)	0.7 (0.4, 1.3)	0.3	0.8 (0.4, 1.5)	0.5
***Gender*, *n (%)***						
Male	52 (85.2)	424 (77.8)	1.0		1.0	
Female	9 (14.8)	121 (22.2)	0.6 (0.3, 1.3)	0.2	0.6 (0.3, 1.3)	0.2
***Ribavirin*, *n (%)***						
Not used	27 (44.3)	248 (45.5)	1.0		1.0	
Used	34 (55.7)	297 (54.5)	1.0 (0.6, 1.8)	0.8	1.1 (0.6, 1.8)	0.8
***HCV Genotype*, *n (%)***						
1,2,4,other	44 (72.1)	439 (80.6)	1.0		1.0	
3	17 (27.9)	106 (19.4)	1.6 (0.9, 2.9)	0.1	1.3 (0.7, 2.4)	0.5
***Sub-optimal DAA*, *n (%)***						
No	44 (72.1)	467 (85.7)	1.0		1.0	
Yes	17 (27.9)	78 (14.3)	2.3 (1.3, 4.2)	0.007	2.2 (1.1, 4.2)	0.02
***CD4 count nadir*, *cells/mm3*, *n (%)***						
0–100	15 (24.6)	144 (26.4)	1.0		1.0	
101+	46 (75.4)	401 (73.6)	0.9 (0.5, 1.7)	0.8	0.9 (0.5, 1.7)	0.8
***Decompensated cirrhosis*, *n (%)***						
No	51 (83.6)	494 (90.6)	1.0		1.0	
Yes	10 (16.4)	51 (9.4)	1.9 (0.9, 4.0)	0.09	1.8 (0.8, 4.0)	0.1
***Diabetes*, *n (%)***						
No	53 (86.9)	498 (91.4)	1.0		1.0	
Yes	8 (13.1)	47 (8.6)	1.6 (0.7, 3.6)	0.2	1.4 (0.6, 3.2)	0.4

Adjusted OR: adjusted for all factors examined in table.

In an alternative analysis using the approach ITT switch = failure analysis, 11 DAA interruptions were counted as failure for a total of 61/606 (10.3%) treatment failures: 50 with non-SVR12 like in the previous analysis plus the 11 who had suspended treatment prematurely (4 for DAA toxicity 3 for patients’ choice, 2 unknown reasons, 1 drug-to-drug interactions, and 1 death, Fig 3B). Predictors of treatment failure were the same as for virological failure (Table 5B).

## Discussion

In this large cohort of HIV-HCV coinfected individuals seen for care in Italy we showed a relatively low rate of DAA initiation and a high rate of cure, also in advanced stages of HCV liver disease.

Our main consideration is that of 2,607 persons who needed to be cured, only less than half (1,090/2,607; 41.8%) were eligible to DAA reimbursement, and only 35.3% (920/2,607) were actually treated. In 595 (22.8% of the total), i.e. those reaching 12-week follow-up after EOT, we observed a success rate of 90%. So National Health System reimbursement was the main driver of treatment access in HIV-HCV as expected.

When restricting to people eligible for reimbursement of treatment, by 2 years from enrolment approximately 70% of patients had access to DAA. Older age and HIV-RNA≤50 copies/mL were associated to faster DAA initiation, higher CD4 count with delayed DAA initiation. These findings might be explained by the fact that longer HIV and HCV infections occur in older people and with lower CD4 counts; among these individuals DAA was reserved to those with virologically controlled HIV disease supposed to be more compliant also with DAA. Individuals harbouring HCV-genotype 3 had a delayed access to DAA consequent to the only recent availability of DAA regimens effective on this genotype.

There are differences across Europe regarding the prioritization of DAA initiation; for example in the Netherlands, there is no limitation to access to DAA and higher percentages of men sex with men (MSM) with less advanced liver disease have been treated compared to Italy; however, the rate of HCV cure in this population has been only slightly greater (80%) than in those with advanced disease (70%) [19]. Of interest, in our analysis, the percentage of treatment success was only slightly lower in people with stiffness >10 kPa (89%) vs. those with 7–10 kPa (95%) or with <7 kPa (96%) although the association was not significant (p = 0.13). More studies need to be conducted to test what is the best prioritization strategy in the presence of an epidemic emergency such as this even in countries with free access to therapy. The issue is further complicated by the fact that universal treatment might also reduce the number of new HCV infections and this is what the Dutch researchers have demonstrated so far [20]. Of note, the majority of the states in the USA have adopted the same strategy for access to DAA which was adopted in Italy.

Overall, in the 595 patients who started DAA and received a full course of therapy the rate of SVR12 was very high, at 92%. This high percentage of success, very close to that observed in clinical trials, could not be predicted at the outset because our population was unselected and enriched with people with advanced liver disease [21].

The only factors associated with the risk of treatment and virological failure were use of suboptimal DAA. Patients with decompensated cirrhosis showed a nearly double risk of failure compared to patients with advanced fibrosis, consistent with the findings of clinical trials [4–8; 22, 23].

It is interesting to note that, in our analysis, genotype 3-infected individuals, if treated with optimal DAA regimen, showed the same rate of success than that of individuals infected by other genotypes, suggesting that also individuals with difficult-to-treat genotypes might achieve eradication with DAA in real-life using a schedule such as SOF+DCL+RBV for 24 weeks that has never been verified in a controlled study.

Our data confirm in a larger setting the observations of a French cohort [24], where the rate of virological success in 189 co-infected individuals with cirrhosis was 93.3%; the authors did not find any associations between suboptimal regimens and risk of failure but it is possible that their analysis was underpowered.

More than 60% of the patients have been treated for more than 12 weeks even in combination with RBV. Treatment duration longer than 12 weeks was more frequent in HCV-genotype 3 (87.3%) and in decompensated cirrhosis (82.8%). This suggests that presumed treatment efficacy rather than schedule simplicity could be the main drivers of the choice of treatment schedule. Our data confirm also the transient lymphopenic effect of RBV resulting in a transient lower CD4 counts at EOT returning to normal after RBV completion [25].

ART was switched before DAA in 28% of the patients, mainly for concerns on interactions; actually 36% of patients on PI-r regimens switched to other regimens. The availability of INI as RAL or DTG allowed the formulation of effective anti-HIV regimens even in patients with long history of anti-HIV therapy resulting in the possibility to effectively treat HCV without interactions with ART [15, 16].

Our study has some limitations; first of all it is observational and therefore unmeasured confounding cannot be ruled out; we do not routinely collect ART adherence data in our cohorts; finally not for all patients included we had week 12 virological data.

In conclusion: despite high SVR12 rates, only 21% (545/2,607) of our HIV-HCV co-infected patients are currently cured. These data document the gap between universal access and effective treatment in Italy, caused by economic limitations, a low capability of treatment centres and limited availability of optimal treatments. Some of these are unlikely to be unique to the Italian setting. In our unselected population, HIV-HCV co-infected patients show a very high success-rate on DAA even those with advanced liver disease and difficult to treat genotypes. The possible impact of prioritization reimbursement policies in different countries needs to be assessed using simulations and stochastic modelling.

## Supporting information

S1 Table List of Ethic Committees that approved the study. (DOCX)

S2 Table Relative hazards of starting DAA in people with stiffness measure available. (DOCX)

S3 Table Relative hazards of starting DAA in patients eligible to reimbursement after June 2015. (DOCX)
