# Supplementary material for: Access and response to direct antiviral agents (DAA) in HIV-HCV co-infected patients in Italy: Data from the Icona cohort
Source: PLoS One. 2017 May 17;12(5):e0177402. doi: 10.1371/journal.pone.0177402 (PMC5435319; doi:10.1371/journal.pone.0177402)
Supplement: S1 Table — (DOCX) [file pone.0177402.s001.docx]

**Table S1. List of Ethic Committees that Approved Icona Study**

| Comitato Etico Locale per la Sperimentazione Clinica dell'Azienda Ospedaliera Luigi Sacco - Via G.B. Grassi,74 - 20157 Milano |
| --- |
| Comitato di Bioteica della ASL Sassari - Via Monte Grappa, 82 - 07100 Sassari |
| Comitato di Bioteica dell'Azienda Ospedali Riuniti di Bergamo - Largo Barozzi,1 - 24128 Bergamo |
| Comitato Etico dell'Azienda Osedaliero Universitaria di Cagliari - Via San Giorgio,12 - 09124 Cagliari |
| Comitato Etico locale dell'Azienda USL 10 di Firenze - Ospedale Pietro Palagi - Viale Margherita,41 50125 Firenze |
| Comitato Etico dell'Azienda Ospedaliera Universitaria "San Martino" di Genova - Largo Rossana Benzi,10 - 16132 Genova |
| Comitato Etico Palermo 1 - AOUP Policlinico Giaccone - Via del Vespro, 129 - 90127 Palermo |
| Comitato Etico dell' Azienda Ospedaliera-Polo Universitario San Paolo - Via A. di Rudinì,8 - 20142 Milano |
| Comitato Etico dell'Azienda Ospedaliera San Gerardo di Monza - Via Pergolesi,33 - 20052 Monza |
| Comitato Etico Locale per la Sperimentazione Clinica dei Medicinali dell'Azienda Ospedaliera Universitaria Senese di Siena - Le Scotte - 53100 Siena |
| Comitato Etico della Fondazione Centro San Raffaele del Monte Tabor - Istituto Scientifico Ospedale San Raffaele - via Olgettina, 60 - 20132 Milano |
| Comitato Etico dell'Azienda Ospedaliera Osepdale Niguarda Ca' Granda - Piazza Ospedale Maggiore, 3 - 20162 Milano |
| Comitato etico di Area Vasta Romagna ed IRST - Viale Ghirotti, 286 - 47023 Cesena |
| Comitato Etico per la Sperimentazione Clinica della Provincia di Vicenza - Via Ridolfi, 37 - 36100 Vicenza |
| Comitato Etico dell'Ente Ospedaliero Osepdali Galliera di Genova - Via delle Mura Cappuccine, 14 - 16128 Genova |
| Comitato Etico per la Sperimentazione Clinica dei Medicinali della Provincia di Venezia - Via Don Federico Tosatto, 147 - 30174 Venezia |
| Comitato Etico dell'Azienda USL di Latina - Via Canova,2 - 04100 Latina |
| Comitato Etico Indipendente dell'IRCCS CRO di Aviano - Via F. Gallini, 2 - 33081 Aviano (PN) |
| Comitato Etico Unico per la Provincia di Parma - Via Gramsci, 14 - 43100 Parma |
| Comitato Etico Provinciale per la Sperimentazione Clinica - Viale Tre Martiri 45100 Rovigo |
| Comitato Etico dell'Azienda Ospedaliero Universitaria Santa Maria della Misericordia di Udine - Via Colugna, 50 - 33100 Udine |
| Comitato Etico dell'Azienda Ospedaliera Spedali Civili di Brescia - Piazza Spedali Civili, 1 - 25123 Brescia |
| Comitato Etico dell'Azienda Ospedaliera "Istituti Ospitalieri" di Cremona - Viale Concordia,1 - 26100 Cremona |
| Comitato di Bioetica della Fondazione IRCCS Policlinico "San Matteo" - Viale Golgi, 19 - 27100 Pavia |
| Comitato Etico Interaziendale delle AA.SS.LL di Torino - Strada dell'Arrivore 25/A - 10154 Torino |
| Comitato Etico dell'Azienda Ospedaliera "Ospedale di Lecco" - Via dell'Eremo 9/11 - 23900 Lecco |
| Comitato Etico dell'Azienda Ospedaliera Ospedale di Circolo di Busto Arsizio - Piazzale Solaro,3 - 21052 Busto Arsizio (VA) |
| Comitato Etico Indipendente dell'Azienda Ospedaliera-Universitaria di Bologna, Policlinico S.Orsola-Malpighi - Via Massarenti, 9 - 40138 Bologna |
| Comitato Etico Provinciale di Reggio Emilia - Viale Risorgimento 57 - 42100 Reggio nell' Emilia |
| Comitato Eitico per Sperimentazione Clinica della Provincia di Padova - Via Giustiniani, 1 - 35128 Padova |
| Comitato Etico Provinciale di Modena - Via del Pozzo 71 - 41100 Modena |
| Comitato Etico Provinciale di Ferrara - Corso Giovecca, 203 - 44100 Ferrara |
| Comitato Etico dell’Azienda Ospedaliero-Universitaria Ospedali Riuniti Umberto I- G.M. Lancisi - G. Salesi di Ancona – Via Conca 71 – 60126 Ancona |
| Comitato Etico Interzonale della ASUR zona Territoriale 8 di Civitanova Marche e Zona Territoriale 9 di Macerata - Via Santa Lucia 2 - 62100 Macerata |
| Comitato Etico per la Sperimentazione Clinica dei Farmaci della AUSL di Pescara - Via Fonte Romana, 14 - 65100 Pescara |
| Comitato Etico dell'Università Cattolica del Sacro Cuore - Policlinico Universitario Agostino Gemelli di Roma - Largo A. Gemelli, 8 - 00168 Roma |
| Comitato Etico dell'IRCCS Istituto Nazionale per le Malattie Infettive "Lazzaro Spallanzani" di Roma - Via Portuense, 292 - 00149 Roma |
| Comitato Etico per la Sperimentazione dei Medicinali dell'azienda Ospedaliera-Universitaria Careggi di Firenze - Viale Pieraccini, 28 - 50139 Firenze |
| Comitato Etico delle Aziende Sanitarie dell'Umbria di Perugia - Via della Rivoluzione, 16 - 06070 Perugia |
| Comitato Etico per le Attività Biomedichedell'Università degli Studi "Federico II" di Napoli - Via Pansini, 5 - 80135 Napoli |
| Comitato Etico dell'azienda Policlinico Umberto I di Roma - Viale del Policlinico, 155 - 00161 Roma |
| 3 RomaComitato Etico Indipendente dell'Azienda Ospedaliera Universitaria Policlinico Tor Vergata di Roma - Viale Oxford, 81 - 00133 Roma |
| Comitato Etico Centrale IRCCS Lazio-Sezione IRCCS IFO-Fondazione GB Bietti - Via Elio Chianesi, 53 - 00144 Roma |
| Comitato Etico Lazio 1 - Circonvallazione Gianicolense, 87 - 00152 Roma |
| Comitato Etico dell'Università "Sapienza" - Viale del Policlinico 155 - 00161 Roma |
| Comitato Etico delle Aziende Sanitarie della Regione dell'Umbria - Via della Rivoluzione, 16 - 06070 Ellera di Corciano (PG) |
| Comitato Etico Indipendente Locale dell'Azienda Ospedaliera Ospedale Policlinico Consorziale di Bari - Piazza Giulio Cesare,11 - 70124 Bari |
| Comitato Etico dell'Azienda Ospedaliera Domenico Cotugno di Napoli - Via G. Quagliariello, 54 - 80131 Napoli |
| Comitato di Etica per la Ricerca Biomedica dell'Università degli Studi "G. D'annunzio" e della ASL di Chieti - Via dei Vestini, 31 - 66013 Chieti |
| Comitato Etico Catania 2 - Piazza S. Maria di Gesù, 7 - 95123 Catania |
| Comitato Etico di Messina - Via Consolare Valeria,1 - 98125 Messina |
