# Supplementary material for: Access and response to direct antiviral agents (DAA) in HIV-HCV co-infected patients in Italy: Data from the Icona cohort
Source: PLoS One. 2017 May 17;12(5):e0177402. doi: 10.1371/journal.pone.0177402 (PMC5435319; doi:10.1371/journal.pone.0177402)
Supplement: S2 Table — (DOCX) [file pone.0177402.s002.docx]

**Table S2. Relative hazards of starting DAA in people with stiffness measure available**

|  | **Relative hazards of starting DAA (only people with stiffness measure available)** | | | |
| --- | --- | --- | --- | --- |
|  | **Unadjusted HR (95% CI)** | **p-value** | **Adjusted HR (95% CI)** | **p-value** |
| ***Age, years*** |  |  |  |  |
| >50 years vs. below | 1.4 (1.1, 1.7) | 0.001 | 1.4 (1.1, 1.8) | 0.006 |
| ***Gender*** |  |  |  |  |
| Female vs. Male | 1.0 (0.8, 1.3) | 0.6 | 1.1 (0.9, 1.5) | 0.3 |
| ***Mode of HIV Transmission*** |  |  |  |  |
| Heterosexual contacts | 1.0 |  | 1.0 |  |
| IDU | 1.0 (0.7, 1.3) | 1.0 | 1.1 (0.8, 1.6) | 0.4 |
| Homosexual contacts | 1.0 (0.6, 1.7) | 0.8 | 1.2 (0.7, 2.2) | 0.5 |
| Other/Unknown | 1.4 (0.9, 2.1) | 0.1 | 1.5 (0.9, 2.6) | 0.1 |
| ***Employment*** |  |  |  |  |
| Unemployed | 1.0 |  | 1.0 |  |
| Employed | 1.1 (0.9, 1.5) | 0.3 | 1.2 (0.9, 1.6) | 0.3 |
| Other/unknown | 1.3 (0.9, 1.8) | 0.2 | 1.2 (0.8, 1.9) | 0.3 |
| ***CD4 count, cells/mm3*** |  |  |  |  |
| per 100 higher | 1.0 (1.0, 1.0) | 0.07 | 1.0 (0.9, 1.0) | 0.02 |
| ***HIV-RNA, copies/mL*** |  |  |  |  |
| 0-50 vs. >50 | 0.9 (0.7, 1.2) | 0.6 | 1.3 (0.9, 1.8) | 0.1 |
| ***Time from HIV diagnosis, years*** |  |  |  |  |
| per 10 longer | 1.0 (0.8, 1.1) | 0.4 | 1.0 (0.9, 1.1) | 0.9 |
| ***HCV genotype*** |  |  |  |  |
| 1a | 1.0 |  | 1.0 |  |
| 1b | 0.8 (0.6, 1.1) | 0.2 | 0.8 (0.6, 1.2) | 0.3 |
| 2 | 1.0 (0.5, 1.8) | 0.9 | 0.7 (0.3, 1.6) | 0.3 |
| 3 | 0.9 (0.7, 1.1) | 0.2 | 0.8 (0.6, 1.00) | 0.05 |
| 4 | 1.0 (0.7, 1.2) | 0.7 | 1.0 (0.7, 1.4) | 0.9 |
| Other/unknown | 0.8 (0.4, 1.7) | 0.6 | 0.8 (0.3, 2.4) | 0.7 |
| ***HCV-RNA, log10 IU/l*** |  |  |  |  |
| per log higher | 0.9 (0.8, 1.0) | 0.1 | 0.9 (0.8, 1.0) | 0.2 |
| ***Fib4*** |  |  |  |  |
| 0-1.45 | 1.0 |  | 1.0 |  |
| 1.46-3.25 | 1.3 (0.9, 1.7) | 0.1 | 1.2 (0.9, 1.8) | 0.2 |
| 3.25+ | 1.3 (1.0, 1.8) | 0.06 | 1.2 (0.7, 1.8) | 0.5 |
| ***Decompensated cirrhosis*** |  |  |  |  |
| Yes vs. No | 1.2 (0.9, 1.7) | 0.1 | 1.1 (0.7, 1.5) | 0.7 |
| ***Diabetes*** |  |  |  |  |
| Yes vs. No | 1.3 (1.0, 1.8) | 0.08 | 1.2 (0.8, 1.7) | 0.3 |
| ***Platelets,*** |  |  |  |  |
| >150,000 vs. below | 0.8 (0.7, 1.0) | 0.04 | 0.9 (0.7, 1.2) | 0.7 |
| ***ALT, IU/l*** |  |  |  |  |
| >2 ULN vs. below | 1.0 (0.9, 1.2) | 0.6 | 1.2 (0.9, 1.4) | 0.2 |
| ***Bilirubin, IU/l*** |  |  |  |  |
| per 10 higher | 1.0 (0.4, 2.7) | 1.0 | 0.8 (0.2, 2.3) | 0.6 |
| ***eGFR,*** |  |  |  |  |
| 90+ | 1.0 |  | 1.0 |  |
| 60-90 | 1.1 (0.9, 1.4 ) | 0.2 | 1.0 (0.6, 1.5) | 0.9 |
| 0-60 | 0.9 (0.6, 1.3) | 0.5 | 1.1 (0.9, 1.4) | 0.5 |
| ***Previous failure of HCV treatment*** |  |  |  |  |
| Yes vs. No | 1.1 (1.0, 1.4) | 0.29 | 1.1 (0.9, 1.4) | 0.2 |
| Adjusted HR: adjusted for all factors examined in table and stratified by cohort | | | | |
